# Biomimetics Design of Tooth Root Zone at Cylindrical Gears Profile

**DOI:** 10.3390/biomimetics8030308

**Published:** 2023-07-12

**Authors:** Ivana Atanasovska, Dejan Momcilovic, Tatjana Lazovic, Aleksandar Marinkovic, Natasa Soldat

**Affiliations:** 1 Mathematical Institute of the Serbian Academy of Sciences and Arts, 11000 Belgrade, Serbia; 2 Institute for Testing of Materials IMS, 11000 Belgrade, Serbia; dejan.b.momcilovic@gmail.com; 3 Faculty of Mechanical Engineering, University of Belgrade, 11000 Belgrade, Serbia; tlazovic@mas.bg.ac.rs (T.L.); amarinkovic@mas.bg.ac.rs (A.M.); 4 The Academy of Applied Technical Studies, 11000 Belgrade, Serbia; nsoldat@atssb.edu.rs

**Keywords:** biomimetics, cylindrical gears, tooth root design, fatigue resistance, finite element analysis, theory of critical distances

## Abstract

During the last few decades, the requirements for modern machine elements in terms of size reduction, increasing the energy efficiency, and a higher load capacity of standard and non-standard gears have been very prevalent issues. Within these demands, the main goals are the optimization of the gears’ tooth profiles, as well as the investigation of new tooth profile designs. The presented design idea is based on the optimal solutions inspired by nature. Special attention is paid to the new design of the tooth root zones of spur gears in order to decrease the stress concentration values and increase the tooth root fatigue resistance. The finite element method is used for stress and strain state calculations, and the particular gear pair is modeled and optimized for these purposes. For tooth root strength analysis, the estimations are based on the theory of critical distances and the stress gradients obtained through finite element analysis. The obtained stress gradients have shown important improvements in the stress distribution in the transition zone optimized by biomimetics. An analysis of the material variation influence is also performed. Based on the investigations of a particular gear pair, a significant stress reduction of about 7% for steel gears and about 10.3% for cast iron gears is obtained for tooth roots optimized by bio-inspired design.

## 1. Introduction

The requirements for size reduction, energy efficiency, and an increase in load capacity are dominant in the contemporary research of machine elements and systems. Thanks to their compact construction, high capacity and reliability, small mass per unit transmitted power, and small energy losses, gears are still the most commonly used machine elements in the power transmission systems in various industry areas. Therefore, despite the wide base of available research results, new research is necessary in order to improve the tooth profile design and upgrade the load capacity calculation procedures for different real working conditions. This task requires a refinement of the results of extensive theoretical and experimental research, as well as an implementation of new multidisciplinary research and disciplines, such as biomimetics design [1,2,3,4,5] and the design of new or improved materials [6,7,8,9,10]. Few research papers in which the authors used the basic biomimetics principles for the geometrical optimization of standard mechanical components could be found. Zhang et al. [11] proposed an innovative bionic design of a toothed wheel for soil imprinting in an on-farm field and verified the new design through finite element analysis, while Mattheck et al. optimized the screw geometry and compared the optimization performed through the analytical pocket calculator method with one performed using the graphical method of tensile triangles, which significantly reduces the maximum stresses by up to about 34% [12]. During the few last decades, scientists have recognized these postulates and have performed a series of theoretical, experimental, and numerical research in order to obtain an improved tooth profile design, as well as the precise methods and procedures for gears’ load capacity calculations.

The Involute tooth profile defined decades ago, given by standards ISO 53 (1998) [13] and DIN 867 (1986) [14], is still widely used in practice. Different authors have researched possible modifications or optimizations of this basic profile [15]. Wen et al. researched the spur gears with tip relief [16], while Velex, Bruyère, and Gu developed a new approach for optimal profile modifications for high-contact-ratio gears [17]. The verified mathematical optimization methods, such as the differential evolution algorithm and its modifications, are very common methods for the optimization of the parameters of involute tooth profiles [18,19]. From the other side, some of the published research contains very specific new ideas for design modifications, such as the modified curvilinear gear set proposed and analyzed by Yi-Cheng and Chien-Cheng [20]. Also, authors have paid special attention to the design of involute asymmetric gears, e.g., Kapelevich [21] developed the procedure for designing asymmetric gears with parameters independent from the generating rack parameters. With a similar background, Marimuthu and Muthuveerappan [22] investigated the application of the direct gear design approach for the design of involute asymmetric gear pairs, while Pedersen [23] discussed possible improvements to the bending load capacity in spur gears using asymmetric gears and the appropriate tooth rack shape optimization.

In recent years, authors have also recommended new profile designs and discussed the advantages of the proposed designs. A series of research is available for the design modifications of high-contact-ratio gears, e.g., Wang, Ren, and Li [24] proposed a new design for the internal gear for a high-contact-ratio gear pair and discussed the obtained increase in the contact ratio of the novel internal gear drive. Some of the recent advanced results in this field provided cylindrical helical gear drives with a variable helix angle [25] and a novel non-involute design based on the control of relative curvature [26]. Roth, Paetzold, and Roth [27] developed an improved gear root fillet based on the decreasing notch effect that exists in nature (referring primarily to the nature solution of a zone where a tree trunk breaks through the earth) and proposed it as an innovative design [28]. All of these research papers and results encourage further research on tooth profile improvements.

Also, due to the new possibilities for the manufacturing and finishing of gears, a few very interesting research studies of new profile designs have been published. Kuhr [29] published an introduction to gear optimization with a focus on the optimization of plastic gear geometry. More specifically, Koide, Yukawa, Takami et al. [30] investigated the characteristics of plastic sine-curve gears in comparison to standard involute gears, while Zorko et al. [31] discussed the improved characteristics of S-polymer gears manufactured through cutting. These trends, combined with the development of the optimization procedures for standard tooth profiles, offer increasing possibilities for gear transmission system implementations in all industry branches, as well as in all ranges of dimensions, from the micro to macro scale. This returns the focus to developing advanced load capacity calculation procedures for gears without limitations in terms of the dimensions and materials. This task requires refined results of extensive theoretical and experimental research that will help correct the existing analytical procedures, mainly defined by the currently used standards [32,33], and in developing new advanced procedures based on the combination of the verified computationally supported methods, such as finite element analysis and theory of critical distances [34,35].

The operational performances of gear power transmissions primarily depend on the tooth root capacity, i.e., the bending load capacity of gears. The analytical procedures for bending capacity calculations are based on the formulae defined by Niemann [36]. Also, standard procedures like ISO recommendations [33] are still widely used in engineering practice and undoubtedly give enough precise values for the bending load capacity of gears in a certain defined range of sizes and loading conditions, as well as for ones made of traditional materials. However, for gear pairs that operate in strictly defined conditions with high requirements regarding dimensions, reliability, safety, and noise, as well as for gears that are out of the ranges covered by standardized procedures, new specific models and procedures need to be developed. Research in that sense is mainly devoted to the application of finite element analysis (FEA) for individual gear pair cases. In recent years, new trends for improvement in FEA and the development of new methods and procedures are in focus. Therefore, Aziz and Chassapis [37] made an effort to use the principles of the design for reliability approach to develop the stress–strength interference (SSI) theory for detailed gear design, while Kapelevich [38] used FEA as a tool for bending stress balance and minimization. There are also a few groups of researchers who in recent years published different results about the optimization of tooth profile parameters with the aspect of gear bending capacity, which is the main point of research described in this paper. The series of results of Li could not be skipped in this context. One of his articles [39] described FEA for the contact and bending strength of spur gears with modeling errors and modifications which exist in real conditions. Sánchez, Pedrero, and Pleguezuelos [40] incorporated FEA in the procedure developed to evaluate the fatigue tooth-root stress. Pedersen [23] also made a step forward and performed an FEA analysis of asymmetric gears to achieve tooth shape optimization and improved bending strength. Atanasovska et al. [41] also used FEA as a part of the new explicit parametric method (EPM) for tooth profile optimization with pinion and wheel tooth root capacity balancing as the main aspect, and they also provided a comparison of different optimization methods [42].

A tooth root load capacity primarily depends on the design of a tooth root zone and its shape, as well as the geometry of a transition zone between a tooth flank and a tooth root subjected to fatigue [43]. The influence of other parameters, such as material characteristics and manufacturing processes, is also very important for the bending strength [44]. This paper focuses on the design method as well as design process required for the modification of the traditional design of this transition zone, as well as for its optimization based on the biomimetic principles in order to obtain zones with modified stress concentrations. Also, the general methodology for the optimization of geometric parameters in transition zones, based on the FEA and TCD [45], is modified in order to choose the tooth root design with optimal stress gradients respected on fatigue. The procedures and methods developed and described in this paper give a basis for high-quality gear bending capacity calculations and are a part of original extensive research dealing with multidisciplinary assessment and the matching of new methods for the development of new complex procedures.

## 2. Transition Zone Solutions Based on Biomimetics

The idea of imitating nature is as old as human civilization. Engineering solutions based on the solution of nature can be found in archaeological records and artifacts from all great civilizations throughout history. Nevertheless, there are a relatively low number of examples of engineering applications commonly used in everyday life and industry. Reasons for this may lie in the fact that uncritical copying of solutions from nature cannot provide useful applications in the conditions and requirements given by modern industry. However, researchers have recently recognized the potential of nature’s solutions as inspirations, and biomimetics in the last few years have achieved considerable scope as a scientific discipline. Biomimetics can be defined simply as an interdisciplinary approach based on the design inspired by nature. Engineering solutions are successfully obtained in the field of new materials design, as well as in the robotics industry. Unfortunately, despite several attempts, such as a bionic train [46] or a bionic car [47], the new design solutions inspired by solutions that nature made in order to minimize and optimize the stress concentrations, such as different transition zones, are not significantly involved in traditional engineering design solutions. This paper presents an attempt to propose a new design of a transition zone inspired by the solutions available all around us.

**Figure 1:**
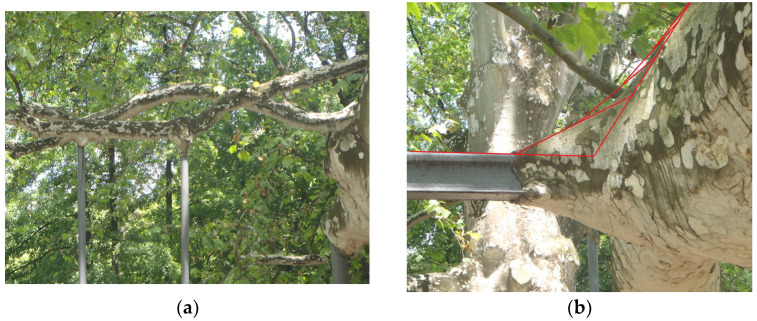
Platanus in Topcider park in Belgrade, Serbia (photo by D. Momcilovic).

**Figure 2:**
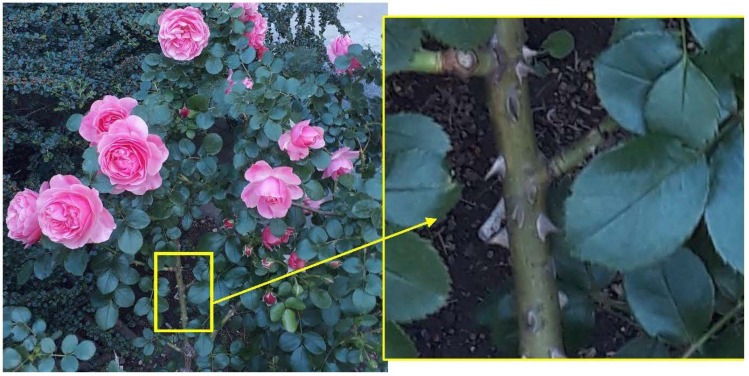
The thorn of the rose in Belgrade, Serbia (photo by I. Atanasovska).

The basis for the presentation of research is the result of Prof. Mattheck [48] and Helms [49] and their good example of solutions offered by nature with emphasis on the design of geometry. Prof. Mattheck researched the transition zones that nature chose and how they are designed on trees. These solutions are optimized to survive even more than a hundred years in different and not always comfortable and pleasant conditions. Mattheck researched the way in which trees grow in order to implement these principles in engineering structures. He found that the transition zone on threes can be mostly explained as a series of isosceles triangles, in which each subsequent triangle has a leg equal to half of the hypotenuse of the preceding one (Figure 1). The transition zones which nature created following the principles based on the series of isosceles triangles discovered by Mattheck are found on the real tree (lat. *Platanus acerifolia*) planted around 1830 and still growing in Belgrade, Serbia (Figure 1). Figure 1a shows a view of this tree from a greater distance, including a few of the points with additional support mounting, which enables us to realize the function of metal bars for supporting its very long branches, as well as shows how the tree has grown up around them, while Figure 1b represents zoomed-in detail of one of the representative transition zones around the supporting bars, how this tree formed during time, with additionally plotted series of Mattheck’s isosceles triangles. It is evident that the theory of the isosceles triangles of Prof. Mattheck gives good simulation of the real transition zone shown in this photo. A similar geometry of the highly loaded transition zone is found on the thorn of the rose, Figure 2, a plant that is also often exposed to variable external conditions for years. The implementation of the above-described principles based on the transition zones inspired by nature was already performed on the shaft to flange transition zone [35]. The investigation led to the conclusion that the biomimetics design gives a significant decrease in the maximum tensile stresses in the critical zones, estimated in the presented case study of the highly loaded turbine shaft to the flange transition zone to an average percentage of 11%.

The operational conditions of spur gears often cause the phenomena of fatigue damage and even failures under these conditions on the tooth roots. Although the tooth root’s bending conditions and the complex loadings of the biomimetics solutions of nature presented in Figure 1 and Figure 2 cannot be analyzed as fully matched, the high level of similarity could be recognized as for both of these biomimetics systems, the failure could be expected as a result of bending conditions (tree’s branches are under bending of their own weight, while the rose thorn is under very high bending loading when it performs its basic function of protecting the rose from predators by stabbing into them).

**Figure 3:**
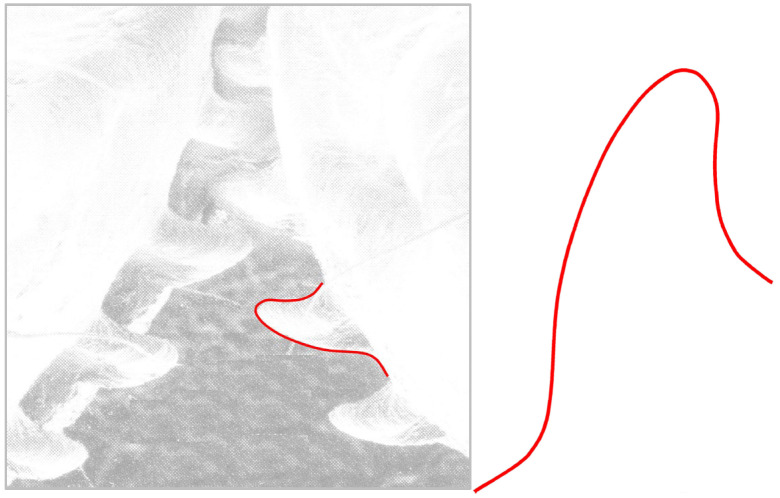
The approximation of tooth profile of the functional gears on the juvenile form of an insect called a planthopper.

In further research of the transition zones calculated by nature, an interesting conclusion is found by comparison of the transition zones created on plants and the approximation which can simulate a tooth root fillet on the functional gears on the juvenile form of an insect called a planthopper (Figure 3). Professors Burrows from the University of Cambridge and Gregory Sutton from the University of Bristol published in the journal *Science* [50] this discovery of the first functional gears in animals. Since then, the photos with the micro-gear pair at the juvenile insect’s hind legs, which help in the synchronization of the legs when the animal jumps, are sharded by and commented on mostly by biologists. In the research we present in this paper, approximation of the curve of the tooth profiles of these gears is performed. The obtained form is presented in Figure 3. It can be concluded that the obtained transition zone in the tooth root is very similar to the solution found in the trees and the roses.

In accordance with this discussion, in the next sections of this paper, the transition zone explained by Mattheck’s isosceles triangles design is used for the new tooth root design. The stresses corresponding to the gears’ bending strength are calculated and compared with the results obtained for the traditional tooth root design.

## 3. Tooth Root Design Based on the Biomimetics Principles

**Figure 4:**
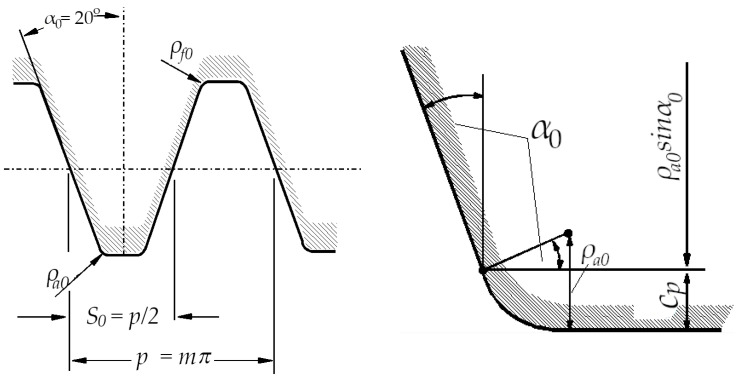
The design of the tooth root zone of standard tooth profile.

**Figure 5:**
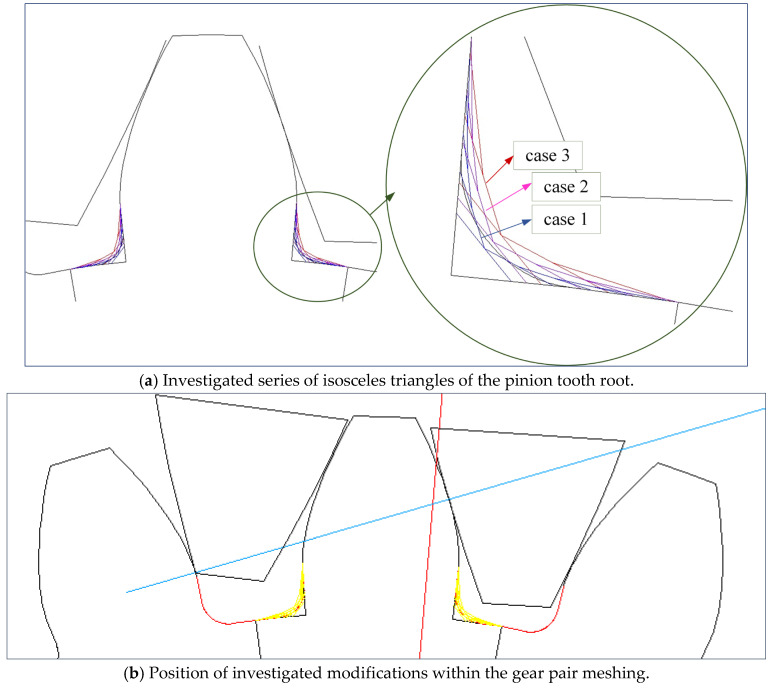
Tooth root with series of isosceles triangles.

In this paper, special attention is paid to the new design of tooth root zones of spur gears to decrease the stress concentration values and increase the tooth bending strength. The starting point for the analysis of the possible modification of transition zones at the tooth profiles for cylindrical gears, based on the biomimetics principles, is the definition of the standard traditional design of the profiles and tooth root transition zones. It is defined in accordance with the standard basic rack tooth profile, based on the ISO standard [13] and DIN standard [14], and is shown in Figure 4. The design characteristics of the transition zone at the standard rack profile tip are used as a design for the tooth root of the reference cases used in the presented analysis, while Mattheck’s principle of a series of triangles is used for designing the modifications (Figure 5).

**Figure 6:**
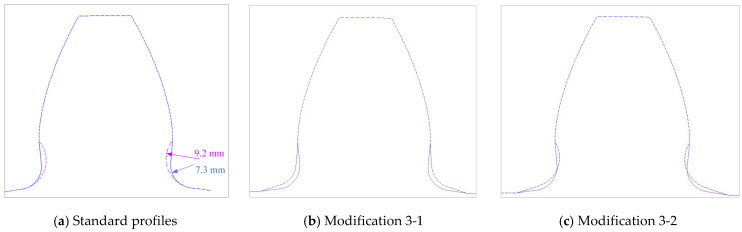
Tooth profiles with standard tooth root design and modified tooth root designs for case 3 triangles.

The presented study is performed for a particular real gear pair [34,42] with the following main dimensional characteristics [51]: number of pinion teeth *z*_1_ = 20, number of wheel teeth *z*_2_ = 96; profile shift coefficients: *x*_1_ = 0, *x*_2_ = 0.328; facewidth *b* = 350 mm; module *m* = *m_n_* = 24; pressure angle *α* = *α_n_* = 20°; contact ratio *ε_α_* = 1.66. In accordance with the standard’s recommendations [13,14], the value of the bottom clearance *c_p_* is chosen to be equal to 20% of the value of the selected standard module. Accordingly, the standard root fillet radius has a value of *ρ_f_* = 7.3 mm (according to the type B tooth profile in ISO 53 [13]). This standard tooth profile is presented with black lines in Figure 5a. For the tooth root standard design in addition to this value, in accordance with DIN recommendations [14], the standard root fillet radius of *ρ_f_* = 9.2 mm is also analyzed. These two standard designs are named “Standard profile 1” and “Standard profile 2” (Table 1). Their design comparison is shown in Figure 6a.

**Table 1:** Description of investigated tooth root design cases.

Design Title	Design Description	Tooth Profile Geometry	Finite Element Model
Standard profile 1	- profile without modifications - root fillet radius: *ρ_f_* = 7.3 mm	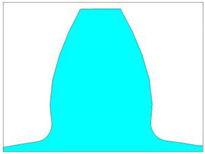	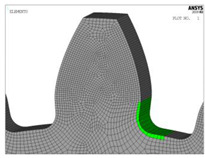
Standard profile 2	- profile without modifications - root fillet radius: *ρ_f_* = 9.2 mm	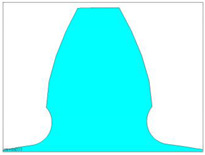	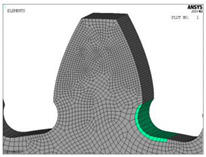
Modification 3-1	- profile with modifications based on the series of triangles case 3 - modification both to the dedendum flank and bottom land surface - root fillet radius: *ρ_f_* = 7.3 mm	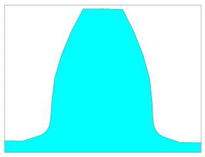	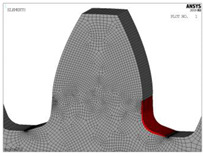
Modification 3-2	- profile with modifications based on the series of triangles case 3 - modification only to the bottom land surface - root fillet radius: *ρ_f_* = 9.2 mm	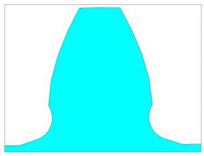	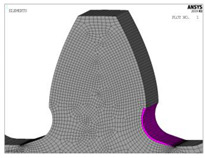
Modification 2-1	- profile with modifications based on the series of triangles case 2 - modification both to the dedendum flank and bottom land surface - root fillet radius: *ρ_f_* = 7.3 mm	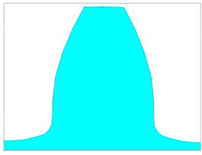	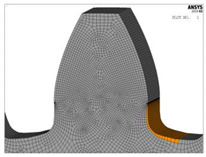
Modification 2-2	- profile with modifications based on the series of triangles case 2 - modification both to the dedendum flank and bottom land surface - root fillet radius: *ρ_f_* = 6 mm	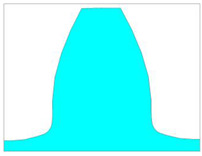	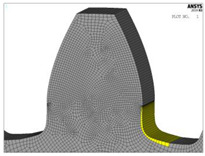
Modification 1	- profile with modifications based on the series of triangles case 1 - modification both to the dedendum flank and bottom land surface - root fillet radius: *ρ_f_* = 7.3 mm	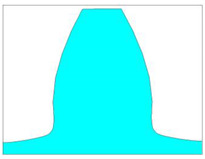	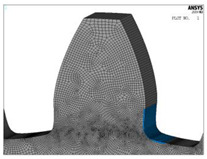

For a new tooth root design inspired by biomimetics, the principle of the creation of generatrixes in the transition zone by a series of isosceles triangles is used. The three series of triangles are created and marked as case 1 (blue line in Figure 5a), case 2 (magenta line in Figure 5a), and case 3 (red line in Figure 5a). The dimensions of these triangles are chosen to start with the biggest one (case 3), for which hypotenuse divides a rounded tooth root zone (with a standard fillet radius of 7.3 mm) in half. The other two cases are based on the smaller triangles, one in front of the fillet radius (case 2—magenta line, Figure 5a), and the other behind the fillet radius (case 1—blue line, Figure 5a). Additionally, in the fillet zones of the modifications, appropriate rounding is applied. The fillet radius is varied with three values: 6 mm, 7.3 mm, and 9.2 mm. The modifications in comparison with the standard tooth profile shown in Figure 5b (standard profile is shown with a red line and all of the investigated modifications are shown with yellow lines) gives a clear representation that the modification edges are very far away from the undercutting conditions; therefore, they could be analyzed as functionally valid shapes. In total, for the presented research, two standard and five modified tooth profiles are analyzed. Their design descriptions, geometries, as well as the corresponding developed finite element models are shown in Table 1. These designs are used in the presented research for bending strength assessment and discussion.

## 4. Methods and Methodology for Bending Strength Assessment

**Figure 7:**
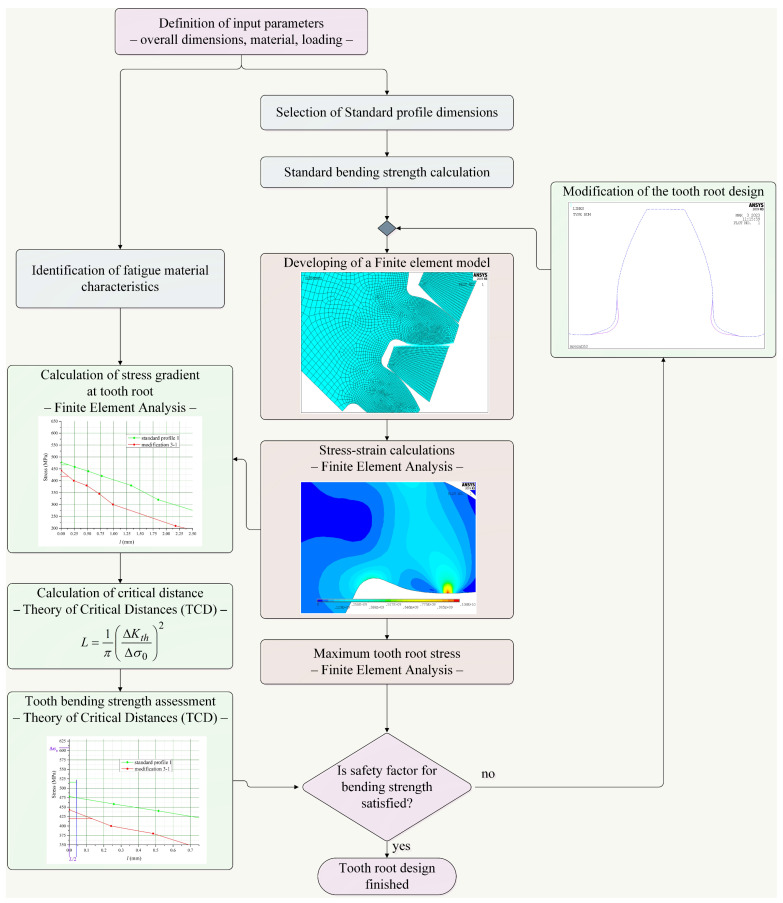
Biomimetics optimization of tooth root based on the bending strength assessment.

The bending strength of the tooth profile with the standard design, as well as the design modified by biomimetics principles, is estimated using a new methodology developed by modification of the general methodology for the optimization of geometric parameters in transition zones based on finite element analysis (FEA) and theory of critical distances (TCDs) [34,46,52]. This methodology implemented both of the standard procedures defined by the ISO series [33], as well as the newly developed approach, and can be successfully used for the selection of the tooth root design with optimal stress gradients respected on fatigue. In Figure 7, the developed methodology is presented by an illustrated algorithm and explains all of the procedures and connections within this methodology. The new approach within this methodology is based on two methods:

- The finite element method for stress–strain calculations and determination of stress gradients in the toot root transition zones;
- The theory of critical distances (TCDs) for an assessment of the bending strength of the investigated transition zones.

**Figure 8:**
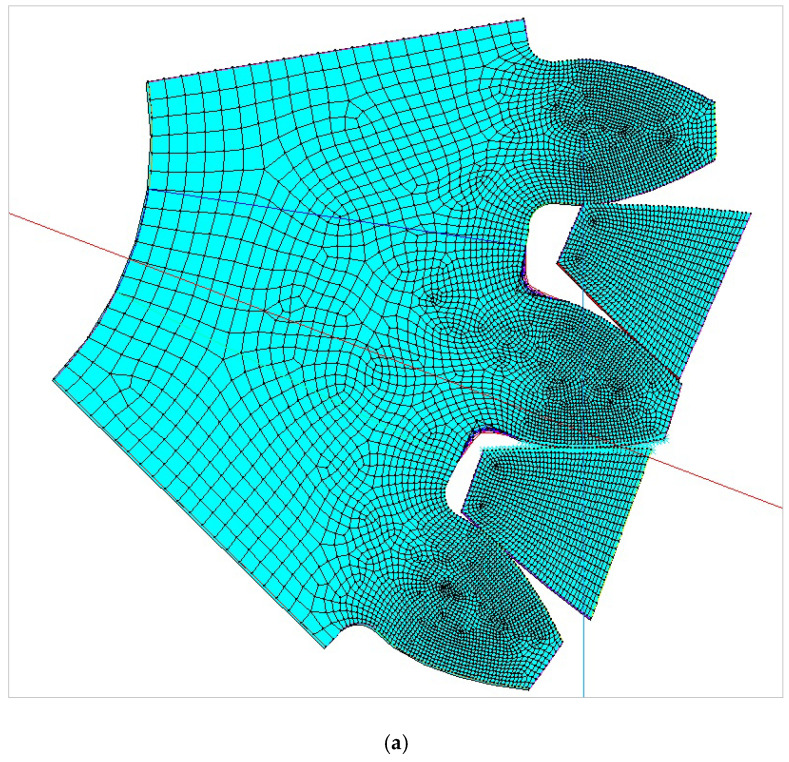

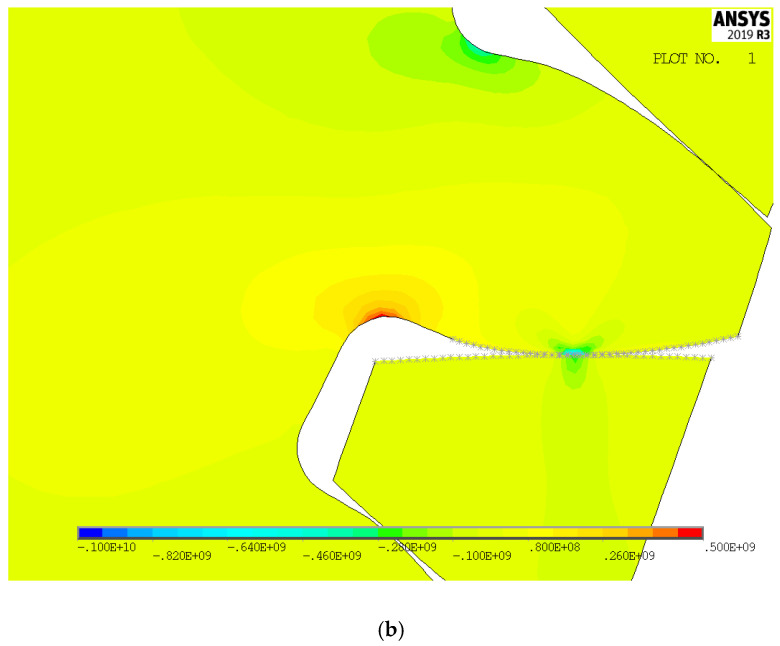
Finite element model and FEA results for normal stresses for the “Standard profile 1” design.

The models for finite element analysis are developed in accordance with the 2D finite element models for spur gears verified in the previous research [53] in comparison with the experimentally measured bending stresses at the tooth root of the gear pair with the same tooth profile parameters, as well as the equal normal loading [54]. The FEA software ASNYS 2019.R3 Mechanical Academic version is used for these purposes. Examples of the developed model for the case of standard profile 1, Table 1, as well as for the corresponding nominal tensile stresses obtained by FEA are shown in Figure 8. The finite element models are developed as 2D models with a plane stress/thickness option as the uniform distribution of load over the facewidth can be assumed for spur gears [32]. The quadrilateral 2D eight-node structural solid finite elements are used for gear discretization, as well as point-to-surface symmetrical contact elements for simulation of the contact conditions. The values for the nominal tooth root stresses are calculated in accordance with ISO recommendations [33] and correspond to the maximum local principal stresses produced at the tooth root when an error-free gear pair is loaded by the static nominal torque and without any pre-stress conditions (Figure 8b). The values of the output results are given in SI system units. Accordingly, the FEA calculations used in this research are performed for the outer point of single pair contact on the driving gear—defined in ISO [51] as point D on the line of action. The developed 2D finite element model is oriented in a way that the line of action matches with the vertical axis (*y*-axis), Figure 8a, which allows for the precise identification of the positions of the characteristic points on the line of action (points A–E).

**Figure 9:**
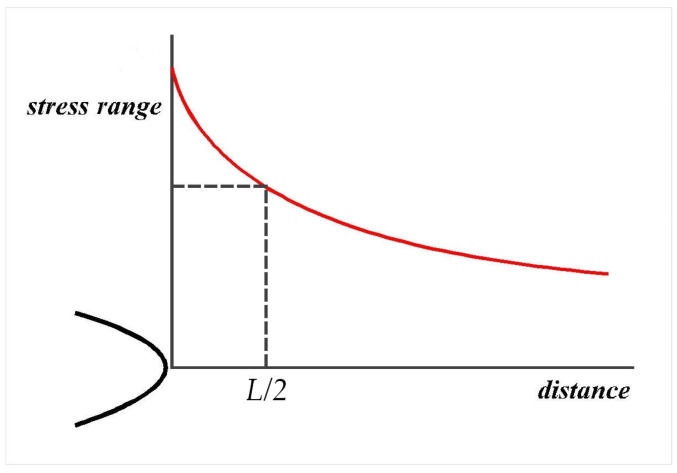
Schematic explanation of the TCD point method.

The part of the presented methodology in Figure 7 which defines the procedure for an assessment of the tooth bending strength for all of the investigated standard designs of the tooth profile, as well as the modified ones, are based on the main postulates of the point method of the theory of critical distance (TCD) [55,56]. The TCD method can be simply presented by an algorithm, given in Figure 9, and is actually a group of methods for failure prediction of various types of stress concentration features on machine elements and other engineering structures, such as contact, sharp and blunt notches, transition zones, and even cracks.

The TCD theory is based on the postulate that fatigue failure consists of the crack initiation and crack propagation up to the moment of failure. In this chain of events, both the maximum stress and the stress gradient determine whether a failure occurs or not. According to the core idea of TCD, all materials possess inherent material length related, in a complex way, with the microstructure and mechanism of deformation. Comparison of this inherent length with the FEA-calculated stress gradient, i.e., finding the intersection point, determines the conditions under which the crack will occur from the considered combination of stress and particular geometry of the stress concentration feature.

The presentation of local stresses around a stress concentration in a simple form by a diagram of the stress as a function of the distance from the stress concentration feature is the main simplification of the point TCD method suitable for wide engineering applications. The major assumption is that the stress analysis is an elastic one, and the FEA is accepted as the most appropriate method for the calculation of these stress gradients. Therefore, the main steps for the application of TCD are as follows:

1. Generating stress—distance diagram, as one presented in Figure 9 based on the finite element analysis;
2. Calculating the value of critical distance as proposed by Taylor [55,56] for point TCD.

(1) $$L=\frac{1}{\pi }{\left(\frac{∆K_{th}}{∆\sigma_{0}}\right)}^{2}$$

where Δ*σ*_0_ is the fatigue strength of standard smooth specimens of the material, and Δ*K_th_* is the fatigue threshold stress intensity. These material characteristics are given in Table 2 for the materials of the investigated gear pair. Taylor has shown that the above-described material characteristic Δ*σ*_0_ is appropriate for calculations of critical distance value *L* for a wide range of materials [57,58].

## 5. Results and Discussion

An analysis of the gear bending capacity by the methods and procedures given in the algorithm shown in Figure 7 is performed on the particular spur gear pair for the large transport machine described in chapter 3. The calculations are carried out for the standard tooth root designs, as well as for the new tooth root designs according to the modifications explained in Table 1. For the particular gear, pair analysis is performed for the rotational wheel speed of *n*_2_ = 4.1596 min^−1^, and for two materials: steel 17CrNiMo7 and cast iron (material characteristics are taken from the literature [58,59] and given in Table 2). The loading conditions are defined by the values of the wheel torque, i.e., a value of *T*_2_ = 1262.95 kN·m for steel gears, and a value of *T*_2_ = 631.47 kN·m for cast iron gears. All of the finite element models are developed for the reduced facewidth of *b* = 30 mm, assuming the uniform distribution of load over the facewidth [32], based on numerical models verified by experiment within the standard profile gears research [54].

**Table 2:** Material characteristics [58,59].

	*R_p_*_02_ (MPa)	*R_m_* (MPa)	Δ*σ*_o_ (MPa)	Δ*K_th_* (MPa√m)
Steel	1021	1366	612	18
Cast iron	310	445	160	15.9

Based on the data given in Table 2, the values of critical distances for both materials are calculated in accordance with the TCD definition given in Equation (1):

(2) $$L=\frac{1}{\pi }{\left(\frac{∆K_{th}}{∆\sigma_{0}}\right)}^{2}=\frac{1}{\pi }{\left(\frac{18}{612}\right)}^{2}=0.275\;mm-\mathrm{for}\;\mathrm{steel}$$

(3) $$L=\frac{1}{\pi }{\left(\frac{∆K_{th}}{∆\sigma_{0}}\right)}^{2}=\frac{1}{\pi }{\left(\frac{15.9}{160}\right)}^{2}=3.2\;mm-\mathrm{for}\;\mathrm{cast}\;\mathrm{iron}$$

**Figure 10:**
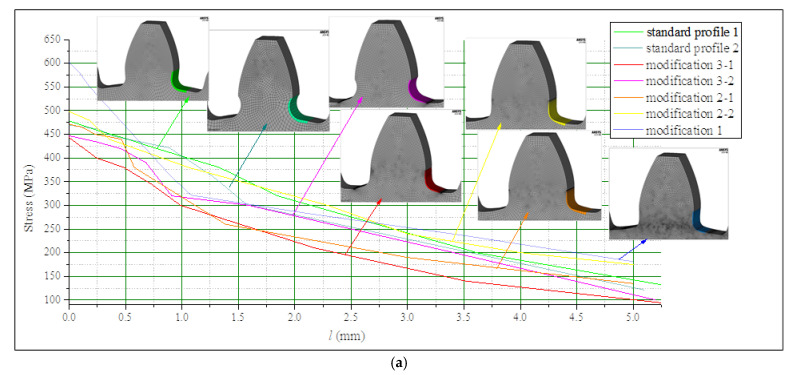

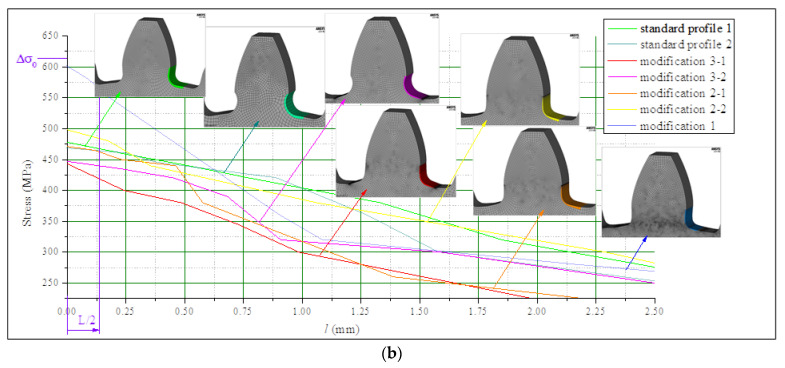
Comparative stress gradient diagrams for steel gears.

**Figure 11:**
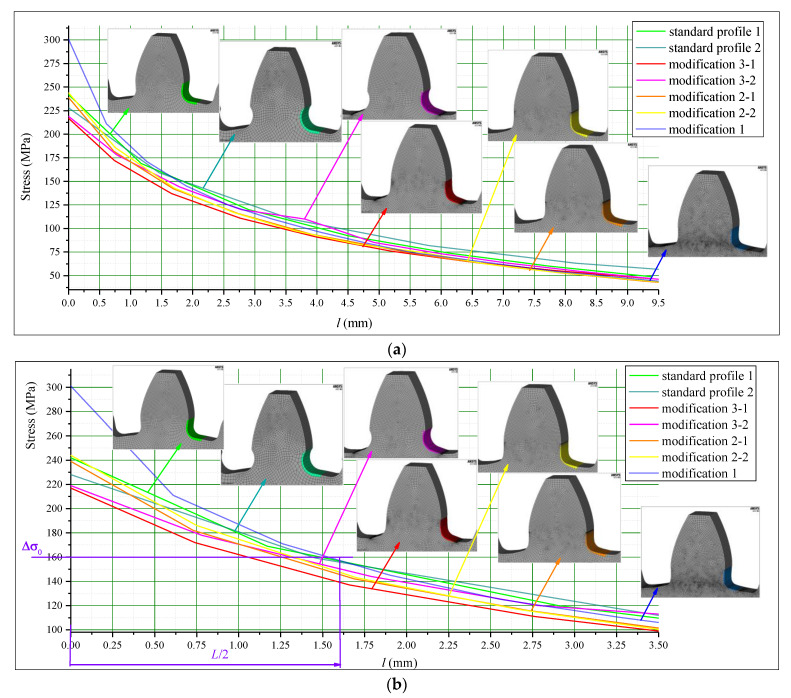
Comparative stress gradient diagrams for cast iron gears.

After performing a set of FEAs for all the investigated cases described in Table 1, and for both the materials and loadings defined above, the normal tensile stress gradients are read out from the obtained FEA results. In accordance with the procedure given in Figure 9, two sets of stress gradient diagrams are created. They are presented by comparative diagrams in Figure 10—for steel gears—and in Figure 11—for cast iron gears. 

In both figures (Figure 10 and Figure 11), the first of the diagrams (Figure 10a and Figure 11a) show the obtained stress gradients starting from the point with maximum bending stresses at the tooth root, up to the distance from this point in which the stresses reach much lower values than the critical ones and are approximately equal for all considered designs. The second diagrams given in these figures (Figure 10b and Figure 11b) show the zoomed-in details with additional displaying of the values of fatigue strength and corresponding calculated critical distances (Equations (2) and (3)).

Based on the analysis of the presented diagrams, it is obvious that some of the investigated modifications give lower values for the maximum tensile stresses at the radius fillet of the tooth root zones, as well as more intense stress reduction along the line normal to the transition surfaces, which actually means higher fatigue strength. Additionally, analyzing the results obtained for the particular investigated cast iron gears gives a conclusion that the solutions obtained for standard proposed designs will not satisfy the safety factors, while few of the new proposed designs provide the required bending capacity.

**Figure 12:**
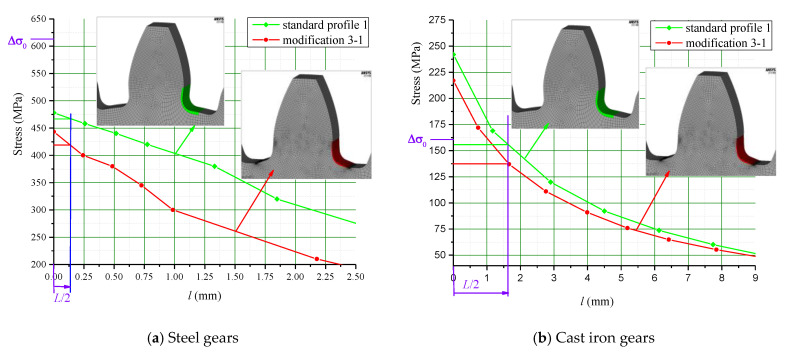
Stress diagrams comparison: “Standard profile 1” design vs. “Modification 3-1” design.

**Figure 13:**
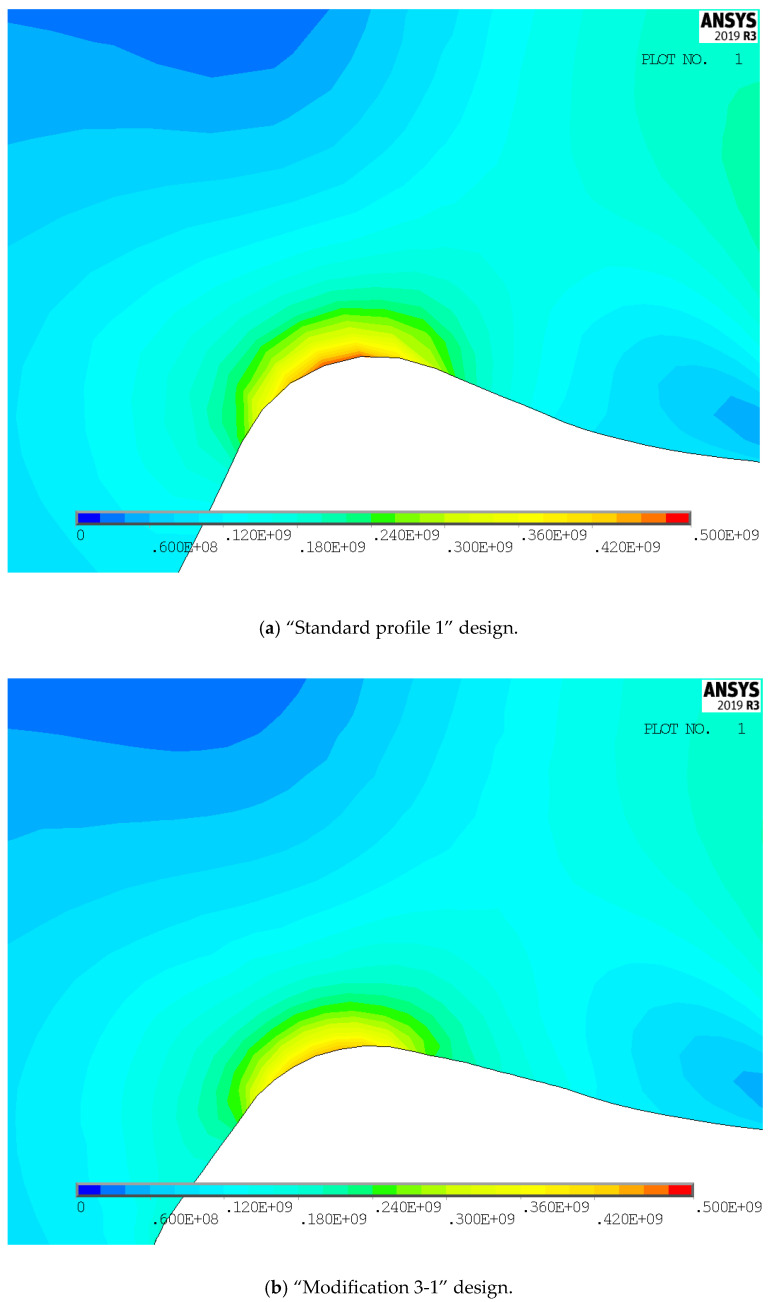
Contour stress plots of the FEA results for normal tensile stresses (in N/m^2^)—standard and modified tooth root design.

For both of the investigated materials, the biggest stress reduction is obtained for the design named “Modification 3-1”, which is created based on the biggest triangles (case 3 in Figure 5) and with an additional fillet radius equal to the standard one (7.3 mm). In comparison with the standard design named “Standard 1”, the obtained stress reduction is about 7% for steel gears (Figure 12a) and about 10.3% for cast iron gears (Figure 12b). The stress gradients obtained by FEA for these design cases for steel gears are presented with contour stress plots in Figure 13, giving a very good visualization of the previously explained analysis and conclusion. The values of the output results are given in SI system units.

## 6. Conclusions

An idea for a new tooth root profile design based on the biomimetics principles is given in this paper to underline this new and prospective future framework for improving the gears’ load capacity. The presented research is a part of the comprehensive investigation performed to estimate the capacity of biomimetics for standard machine element improvements. Based on the biomimetics principles, special attention in the presented research is dedicated to the investigation of modifications and improvements on the tooth root design. 

An important contribution is given to the modification and implementation of a methodology for a tooth bending strength assessment developed previously for gears with standard tooth profiles. This methodology implemented the standard procedures, as well as the newly developed approach based on finite element analysis and theory of critical distances. The special advantage of this methodology is its potential to be successfully used for the optimization of non-standard gear design or the design of gear pairs with dimensions and/or conditions not covered by the adopted standards. The methodology’s output is optimal tooth root design selected from the viewpoint of stress gradients respected on fatigue.

Based on the investigations of a particular gear pair with biomimetics tooth root design, the following conclusions are obtained:

- A significant stress reduction of about 7% for steel gears, and about 10.3% for cast iron gears, is achieved;
- Obtained stress gradients show important improvements in the means of more uniform stress distribution in the transition zone of tooth root designed by biomimetics.

The comparison of the bending strength of the standard and newly proposed biomimetics designs leads to additional conclusions being made:

- Biomimetic principles could be a new inspiration for tooth profile optimization;
- A solution of transition zone stress concentrators inspired by nature can be implemented by a series of isosceles triangles;
- Biomimetic design principles could be implemented in new tooth root design solutions.

Finally, in accordance with the general tendencies toward mass reduction, as well as increasing the load capacity and reliability of power transmission elements, it can be concluded that further investigation in the presented framework is required. The presented case study, as well as the obtained results, point out the need for extensive research in order to make valuable applicable improvements. Simultaneously with the numerical experiments, after detailed analysis of the obtained results, the development of the experimental setup for investigated gear profiles is planned. Extensive numerical experiments will reduce the unnecessary cost and duration of the experiment.

## Data Availability

Not applicable.
